# Modulation of Internal Estimates of Gravity during and after Prolonged Roll-Tilts

**DOI:** 10.1371/journal.pone.0078079

**Published:** 2013-10-10

**Authors:** Alexander A. Tarnutzer, Giovanni Bertolini, Christopher J. Bockisch, Dominik Straumann, Sarah Marti

**Affiliations:** 1 Department of Neurology, University Hospital Zurich, Zurich, Switzerland; 2 Department of Otorhinolaryngology, University Hospital Zurich, Zurich, Switzerland; 3 Department of Ophthalmology, University Hospital Zurich, Zurich, Switzerland; McMaster University, Canada

## Abstract

Perceived direction of gravity, as assessed by the subjective visual vertical (SVV), shows roll-angle dependent errors that drift over time and a bias upon return to upright. According to Bayesian observer theory, the estimated direction of gravity is derived from the posterior probability distribution by combining sensory input and prior knowledge about earth-vertical in a statistically optimal fashion. Here we aimed to further characterize the stability of SVV during and after prolonged roll-tilts. Specifically we asked whether the post-tilt bias is related to the drift pattern while roll-tilted. Twenty-nine healthy human subjects (23-56yo) repetitively adjusted a luminous arrow to the SVV over periods of 5min while upright, roll-tilted (±45°, ±90°), and immediately after returning to upright. Significant (p<0.05) drifts (median absolute drift-amplitude: 10°/5min) were found in 71% (±45°) and 78% (±90°) of runs. At ±90° roll-tilt significant increases in absolute adjustment errors were more likely (76%), whereas significant increases (56%) and decreases (44%) were about equally frequent at ±45°. When returning to upright, an initial bias towards the previous roll-position followed by significant exponential decay (median time-constant: 71sec) was noted in 47% of all runs (all subjects pooled). No significant correlations were found between the drift pattern during and immediately after prolonged roll-tilt. We conclude that the SVV is not stable during and after prolonged roll-tilt and that the direction and magnitude of drift are individually distinct and roll-angle-dependent. Likely sensory and central adaptation and random-walk processes contribute to drift while roll-tilted. Lack of correlation between the drift and the post-tilt bias suggests that it is not the inaccuracy of the SVV estimate while tilted that determines post-tilt bias, but rather the previous head-roll orientation relative to gravity. We therefore favor central adaptation, most likely a *shift* in prior knowledge towards the previous roll orientation, to explain the post-tilt bias.

## Introduction

Accurate spatial orientation relative to gravity is achieved by integrating various sensory signals including those from the vestibular organs (utriculus, sacculus and semicircular canals), skin proprioceptors, vision and joint receptors within the central nervous system in a weighted fashion to obtain an optimal internal estimate of the direction of gravity [1]. These internal estimates can be studied both at the level of brainstem reflexes [as, for example, by measuring compensatory eye torsion while roll-tilting the head, termed ocular counterroll or OCR [2–4]] and at the level of the cortex. While measurements of OCR are technically demanding, perceptual estimates of spatial orientation relative to gravity are more widely available and were successfully implemented in various paradigms including line [subjective visual vertical (SVV)] and rod [subjective haptic vertical (SHV)] adjustments. 

Generally, models simulating internal estimation of the direction of gravity assume that otolith afferents have a constant firing rate over time as long as the head remains in a static upright or roll-tilted position [5–7]. Moreover, these models anticipate that upon return to upright, SVV adjustments are accurate. However findings from vestibular nerve recordings in animals, from brainstem ocular motor reflexes and from behavioral studies in humans put this assumption into question. Fernandez and Goldberg [8] reported decreases in the otolith afferent firing rate for steady-state roll-tilt and centrifugal force paradigms in vestibular nerve recordings in primates. During sustained head roll, the amplitude of OCR has been found to decrease over time [9], which is not surprising as OCR is driven mostly by the otolith organs [10,11]. Behavioral studies investigating prolonged roll-tilt have reported drift of the SVV [12–15] and a bias (deviations of perceived visual vertical towards the previous roll position) upon return to upright after prolonged roll [16], termed *post-tilt bias*. Previous approaches to characterize SVV drifts and post-tilt biases generally assumed that the drift patterns are similar across subjects and did not address individual differences. However, in a recent study focusing on SVV stability while upright, we noted distinct individual drift patterns [17]. The mechanism leading to these differences in drift patterns remains to be determined.

Drifts of torsional eye position and SVV over time may occur due to a shift of the reference or ‚null’ position of the gravity estimating system. Understanding these drifts is important since they will impact the interpretation and modelling of results related to gravity perception. For modelling effects of prolonged roll-tilt on SVV, knowledge of the drift dynamics and their individual range is required, and for simulating the post-tilt bias the decay dynamics (and their inter-individual variability) are essential. Based on the heterogeneity of drift observed in upright position, with mostly increasing adjustment errors over time [17], drifts while roll-tilted may also differ in drift direction and amplitude between individual subjects. We therefore aimed to characterize the stability of internal estimates of the direction of gravity during prolonged roll-tilt positions and upon return to upright position using a psychophysical task. Specifically, we asked how much variability in adjustments over time there is between individual subjects and whether the post-tilt bias and the drift while roll-tilted are correlated, suggesting a common mechanism. Due to its widespread use and comparatively simple implementation we opted for the SVV. The SVV, however, is susceptible to systematic, roll-angle dependent errors, i.e., the graviceptive null as adjusted by a luminous arrow is not aligned with true earth-vertical when the subject is roll-tilted. While roll under-compensation - termed A-effect – is noted at roll-angles larger than 60° [5,18–20], variable and small roll over-compensation – termed E-effect – can be found at roll angles smaller than 60° [20–22]. The origin of the A- and E-effect is most likely central and related to the processing of visual input, as previous studies indicated an accurate percept of vertical for subjective postural horizontal [23] and subjective haptic vertical [24] and horizontal [22]. 

 Based on previous SVV measurements obtained immediately after returning back to upright [16,25–27], we hypothesize that during prolonged roll-tilt the internal graviceptive null is being shifted. Upon return to upright, such a shift will then be reflected in the post-tilt bias of SVV adjustments. In Bayesian modeling an internal estimate of the direction of gravity derived from sensory input is combined with prior knowledge in a statistically optimal way, resulting in a posterior-probability distribution [28–31]. From the posterior probability distribution the brain then selects the maximum a posteriori estimate, i.e. the roll angle with the highest likelihood, which constitutes the internal estimate of the direction of gravity [31]. A shift in the perceived direction of gravity might be driven by adaptation of the peripheral receptors, by central computational mechanisms (e.g. by changing the peak or the width of the probability distribution of prior knowledge about earth-vertical) or by a combination of both peripheral and central mechanisms including random noise. Noteworthy, for generating prior knowledge, the brain integrates peripheral sensory input; therefore changes in the prior may also be driven by peripheral adaptation. For peripheral sensory adaptation, the initial increase (for an excitatory stimulus) or decrease (in case of an inhibitory stimulus) in the afferent nerve firing rate by roll-tilting becomes smaller, reducing the difference between the firing rate while roll-tilted and the normal resting firing rate in upright position [8,32]. This habituation in the firing rate would then be interpreted by the brain as a decrease in the roll-tilt angle. As a result, the subject would under-compensate for roll-tilt when indicating the perceived vertical, i.e. the arrow will deviate towards the subject’s roll-tilt orientation (consistent with an A-effect). Upon return to upright, this peripheral adaptation might persist. However, this has not been systematically studied on the level of vestibular nerve afferent recordings. Conceptually, prior knowledge is thought to reflect perceived direction of gravity as estimated by the brain in the recent past. For this estimate, the CNS integrates the calculated whole-body roll orientation, assuming that small head-tilt angles are most likely. This is reflected by a narrow distribution of the prior that peaks around earth-vertical when the subject is near upright. While this improves the signal-to-noise ratio near upright, taking into account prior knowledge results in systematic errors of perceived direction of gravity for larger whole-body roll-tilt angles. This is because the prior biases the percept of vertical towards the recently experienced roll-angle by shifting the peak of its distribution towards the current roll-tilt position while at the same time the width (variability) of the prior increases. With increasing roll-tilt duration, recent experience about whole-body roll orientation will reflect more and more the current – roll-tilted position and the width of the prior might decrease again. 

Thus, during prolonged roll both the hypothesized habituation in sensory firing rate and prior knowledge could cause a shift in the estimated graviceptive null (as reflected by the posterior distribution derived from the prior and actual peripheral sensory input) towards the subject’s current roll position (i.e. towards the body-longitudinal axis). With such a shift of the graviceptive null during prolonged roll-tilt, subsequent returning to upright position is predicted to result in an initial bias towards the direction of the previous body position. 

To which extent peripheral and central mechanisms contribute to the post-tilt bias, however, is not clear. If the same mechanisms that determine drift during prolonged roll-tilt were also contributing to the post-tilt drift pattern, one would predict similarities in the individual drift patterns, as reflected by significant correlations between the tilt and post-tilt SVV adjustments. Since adaptation at the level of the vestibular afferent firing rates [8,33] during prolonged roll in primates occur, we hypothesize that peripheral sensory adaptation does indeed contribute to SVV drifts during prolonged roll. A significant correlation between the tilt and post-tilt traces would therefore suggest that peripheral adaptation of the same sensors is a relevant contributor to both the drift while roll-tilted and the post-tilt bias. The lack of such a correlation, on the other hand, may indicate either adaptation of other peripheral sensors (not contributing to SVV adjustments during prolonged roll-tilt) or central adaptation (e.g. by shifting prior knowledge) involving distinct mechanisms of adaptation than during prolonged roll-tilt. To test these hypotheses, we searched for individual correlations between adjustment errors in various roll-tilt positions and the subsequent post-tilt bias. 

Significant drifts were found in a majority of runs in roll-tilted positions. When returning upright, an initial bias towards the previous roll-position followed by significant exponential decay was noted in half of all runs. We conclude that the SVV is not stable during and after prolonged roll-tilt and that the direction and magnitude of drift are subject-dependent and roll-angle-dependent. No significant correlations were found between the drift pattern during and immediately after prolonged roll-tilt. We therefore propose that different mechanisms contribute to the drift of the SVV during prolonged roll-tilt and to the post-tilt bias of the SVV. 

## Material and Methods

Twenty-nine healthy human subjects (10 females, 19 males; mean age ± 1 SD: 36 ± 9y) were studied. All subjects completed the SVV recording session, six of the 29 subjects later on also completed a SHV recording session (1 female, 5 males; 39.5 ± 10.1 years old). 

### Ethics statement

Written informed consent of all subjects was obtained after a full explanation of the experimental procedure. The protocol was approved by the local ethics committee (Ethics committee neurology, University Hospital Zurich) and was in accordance with the ethical standards laid down in the 1964 Declaration of Helsinki for research involving human subjects. 

### Experimental setting

All recordings were performed on a motor-driven turntable (Acutronic, Jona, Switzerland). Subjects were secured with a 4-point safety belt with the head restrained in natural straight-ahead position with a thermoplastic mask. Since the otolith organs, which have the greatest contribution to verticality estimation, are situated in the head, the subjects’ orientation in the roll plane will be referred as *head-roll orientation*, although roll movements on the turntable were whole-body. Five head-roll orientations were studied in all subjects (upright, ±45°, and ±90°). These positions were chosen based on previous studies showing substantial A-effects [18] for head-roll angles between 60 and 120-125° and E-effects [21] for head-roll angles < 60° [5,20]. Positions were reached by turntable movements with 10°/s^2^ acceleration and deceleration. We decided to use accelerations and decelerations of 10°/s^2^ since values in this range reflect a compromise between keeping the repositioning time as short as possible and minimizing discomfort of the subject by applying high accelerations and decelerations. These acceleration and deceleration values, however, are well above the detection threshold of the semi-circular canals (SCC) (0.05 °/s^2^ [34,35]) and the perceptual thresholds [36]. Therefore the turntable motion stimuli applied here lead to SCC stimulation and a percept of rotation, which will affect errors in SVV [37,38]. In order to minimize effects of SCC stimulation, the first trial after any chair movement was delayed by five seconds. For static SVV adjustments as used here we have previously checked for post-rotatory torsional ocular drift and nystagmus to quantify the contribution of SCC stimulation after the movement and showed that average torsional eye velocity at the time subjects confirmed arrow adjustments was small (0.10 ± 0.06°/s) [39]. A remote control box allowed the subjects to rotate an arrow (covering the central 9.5° of the binocular visual field) projected on a sphere 1.5m in front and to confirm adjustments. Myopic subjects wore their glasses or contact lenses.

### Experimental paradigm

A single recording session lasting about 60 minutes was completed in all participants. To study effects of adaptation, repetitive SVV adjustments over periods of five minutes in a given roll position were collected. Duration of roll-tilt was limited to five minutes based on previous observations reporting that adaptation occurred mostly during the first three to five minutes [13,14]. At the beginning of each session a five-minute baseline-recording period in upright position was performed. Afterwards the subject was kept in a stationary roll-tilted position for five minutes while repetitively adjusting the SVV before the subject was brought back upright to continue SVV adjustments for another five minutes. A short break with the lights turned on was made at the end of each block while the subject was in upright position, terminating visual adaptation to the dark and allowing the subjects to relax and remove the mask. This procedure was performed for all roll-tilted positions studied. While the first block lasted 15 minutes in total, the subsequent blocks lasted 10 minutes each as baseline recordings were obtained before the first run in roll-tilted position only. Adjustment time for single trials was limited to five seconds and consecutive trials were presented to the subject with a two-second delay. This time limit to complete the task ensured that subjects spent about equal time on the task in all conditions, which reduced potential time-dependent differences in arrow adjustment variability. Completion of each trial had to be confirmed by the subject by pushing a button on the controller. All trials were collected in complete darkness (except for the luminous arrow used to indicate perceived vertical). After changes of turntable roll position, arrow projection started again five seconds after the turntable came to a full stop. The arrow starting roll orientation was random within the entire 360° roll plane for all trials. Before data collection, five to ten training trials were run in each subject. The sequence of roll-positions studied was in random order. The total number of trials varied from subject to subject and depended on the time to complete individual trials. 

As a control experiment in order to study the impact of retinal input on our findings, the same turntable roll positions and recording periods were repeated in six of the subjects that already participated in the SVV paradigm using a non-visual (haptic) paradigm (the subjective haptic vertical or SHV). This second session again lasted about one hour. The starting tube roll orientation was random within a range of ±50° of earth-vertical in all trials. To complete the task, a tactile device (plastic tube, 29 cm long and 2.5 cm thick) was aligned with the perceived direction of gravity in complete darkness using the dominant (right) hand within a time limit of five seconds. This required subjects to grasp this tube with a power grip (large areas of contact between the object and the fingers and palm, little or no ability to impart motions with the fingers) [40] (see also 24 for a detailed description of the experimental setup). 

### Definition of terms frequently used

Clockwise (CW) shifts relative to the earth-vertical axis (as seen by the subject) have positive signs and counter-clockwise (CCW) shifts have negative signs. In the following, we will use the term trial-to-trial variability when we refer to the within-subject median absolute deviation (MAD). In relation to trial-to-trial variability, the term *precision* reflects the inverse, i.e. the degree of reproducibility. *Accuracy*, on the other hand, is defined as the magnitude of the median SVV or SHV adjustment error.

### Data analysis

As our data was not normally distributed (using the Jarque-Bera hypothesis test of composite normality, jbtest.m, Matlab 7.0), non-parametric statistical analyses were applied. This includes non-parametric analysis of variance (Friedman’s test) with multiple comparisons (using Tukey-Kramer to compensate for multiple tests). Data analysis on the group level was obtained using median drift patterns (± one MAD).

#### Quantitative analysis of SVV / SHV drift

 To study the temporal constancy of adjustments during (SVV and SHV) and after (SVV) roll-tilts and to quantify changes, i.e., *drift* over the five-minute recording periods, both linear and exponential functions were fit to the traces. Linear regression analysis using least squares (regress.m, Matlab 7.0, The MathWorks, Nantick, USA) (see Eq. 1) was compared with exponential decay analysis using non-linear least squares (lsqcurvefit.m) (see Eq. 2). 

y=a+b*x (1)

$$y=a*e^{\frac{x}{-Tc}}+c$$ (2)

For both equations the goodness-of-fit (as reflected by the R^2^-value) were obtained and F-tests were used to determine the significance of drift as well as to compare the linear to the exponential fits. Furthermore, Eq. 2 also provided the time constant (Tc) of decay. Goodness-of-fit of the linear and exponential functions across subjects were compared in both roll-tilted and post-tilt conditions using Friedman’s non-parametric ANOVA (friedman.m, Matlab 7.0, The MathWorks). 

Drift patterns were divided into three groups according to their significance (as determined by the F-tests provided by the exponential fits) and direction: a) significant positive (i.e., CW) drift relative to the SVV / SHV settings at the beginning of the five-minute period, b) significant negative (i.e., CCW) drift, and c) non-significant drift. Runs with significant drift were further categorized based on their impact on the size of adjustment errors over time (increasing errors vs. decreasing errors) relative to true earth-vertical. 

We first subtracted individual baseline drift measured in upright position (determined by the exponential fit function) from all post-tilt runs. As a consequence adjustment errors on the post-tilt traces are relative to the subject’s percept of vertical derived from the baseline measurements. This approach takes into consideration individual offsets of perceived vertical (as in the general population SVV is in a range of typically ± 2.5° of true earth-vertical [41,42]). A similar range of It is based on the assumption that baseline drift / fluctuations observed reflect an individually distinct but stable pattern to which a post-tilt drift is added. Recently one of the authors has reported that for serial blocks of repetitive SVV adjustments significant drifts of perceived vertical and horizontal started from similar offset positions and pointed to the same direction in a majority of runs and subjects [43], supporting the assumption that drift patterns are individually stable. 

Furthermore, to characterize the impact of drift on trial-to-trial variability in roll-tilted positions, drift while roll-tilted was determined and removed from the tilt traces for this part of the analysis, comparing trial-to-trial variability in roll-tilted positions with and without drift removed.

#### Correlation analysis

Principal Component Analysis (PCA) was chosen to evaluate correlations between dependent variables. This procedure is equivalent to Orthogonal Linear Regression or Total Least Squares, which minimizes the perpendicular distances from the data points to the fitted model [44]. Least square linear regression differs from PCA in that it implies that the independent variable is known without error. Conversely, PCA adjusts for errors along all axes. We used the R^2^-value as a measure of the goodness of fit. To estimate the sampling distribution of the slope of the fit obtained by PCA, we used bootstrapping to construct 1000 resamples and calculated the 95% confidence interval (CI). A correlation was considered significant whenever the 95% CI of the slope did not include zero.

#### Autocorrelation and spectral density analysis

Previously, we observed that serial SVV or subjective visual horizontal adjustments are not independent [17]. Drifts and fluctuations in SVV estimates may therefore also be related to the self-similarity of consecutive adjustments, that is, consecutive adjustments are dependent and therefore yield similar values, as reviewed by [45]. Based on these considerations, trial-to-trial dynamics for each run were evaluated using spectral density analysis. Generally, consecutive behaviors that show robust serial correlations (reflecting fractal features such as ‘self-similarity’ and ‘scale-invariance’) are considered to be part of a special class known as noise and occur throughout a wide range of different biological systems [45]. Decay of auto-correlations related to 1/*f* ^*β*^ noise was found to be so slow that the generating system is called persistent or long-range dependent [45]. Autocorrelation analysis and spectral density analysis were applied to individual data sets for all conditions and subjects and linear regression analysis was performed to estimate the slope of the fit for spectral density analysis. For a 1/*f* ^*β*^ process the log-log power spectrum is linear with a slope of *β* typically being in the range of -0.5 to -1.5 [45]. According to these authors, consecutive behaviors that are independent yield a slope of 0, while random serial behaviors result in a slope of -2. 

## Results

### Subjective visual vertical paradigm

**Figure 1:**
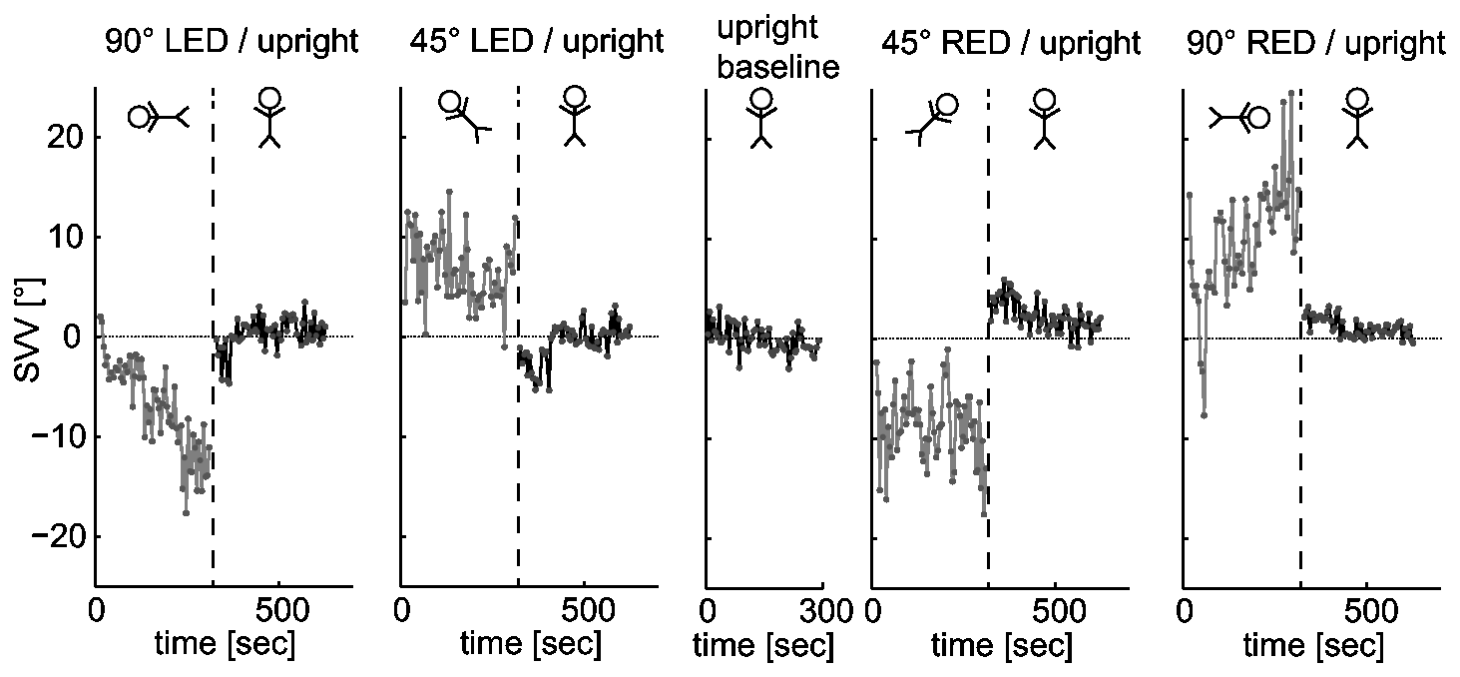
SVV adjustments (filled grey circles) relative to true earth-vertical are plotted against time in a single subject (GB) while roll-tilted (trials interconnected with a grey line) and upon return to upright (trials interconnected with a black line). Baseline recordings (trials interconnected with a black line) of SVV beforehand are shown for comparison. Schematic drawings above the data sets illustrate the subject’s roll orientation as seen from behind. The dashed vertical lines separate sequences with distinct whole-body roll orientations. The dotted horizontal lines indicate true earth-vertical.

**Figure 2:**
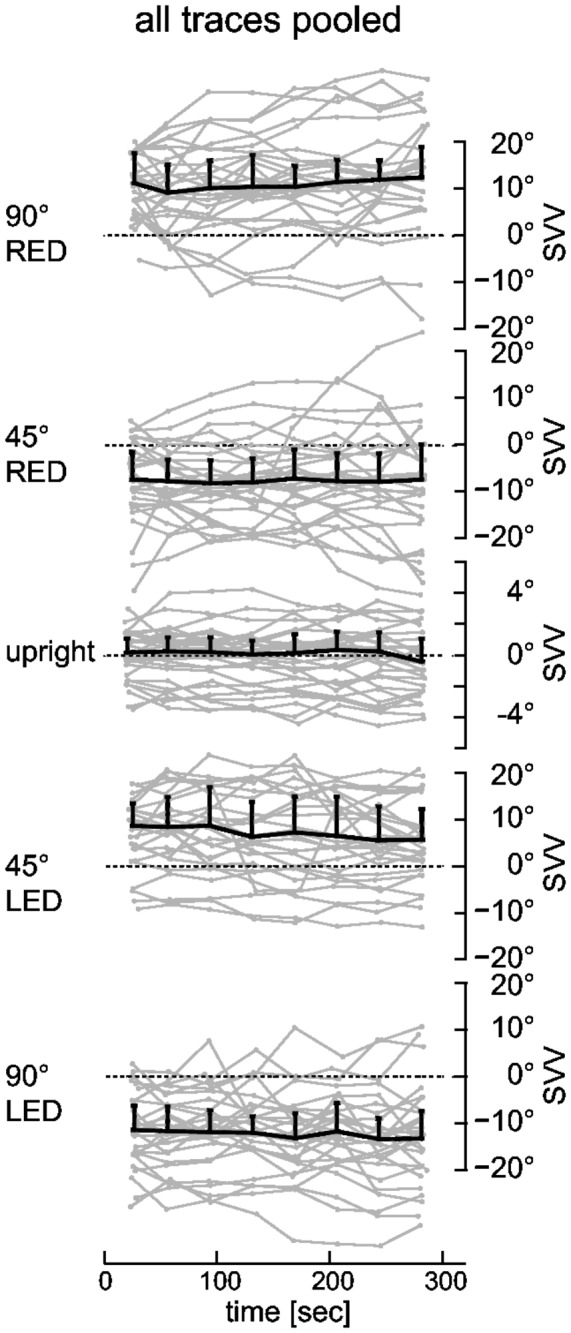
Individual (in grey) and median (in black) adjustment errors while roll-tilted are plotted against time. To improve the illustration of drift, single adjustments were assigned to one of eight bins of equal length and the median (± one MAD) was calculated for each bin. To demonstrate the overall behavior of our study group adjustments from all 29 subjects are pooled. This also allows a comparison with findings from previous studies that did not separate traces based upon their drift characteristics. The dashed horizontal lines refer to perfectly accurate (earth-vertical) adjustments. Note that for baseline upright trials the scaling along the y-axis (as indicated always on the right side of each plot) differs from the roll-tilted positions.

A median of 55 trials (± 3; one MAD) was completed within the five-minute recording periods over the entire study population. Figure 1 illustrates drift of the SVV adjustments (raw data) at baseline, while roll-tilted and immediately upon return to upright position in a typical subject. When pooling the SVV data from all subjects, median drift amplitudes were small (see Table 1 for exact numbers) and not significantly different from zero (non-parametric signtest.m, p>0.05) at either ±45° or ±90°, i.e., SVV settings remained stable over time at the group level (Figure 2) during prolonged static roll. 

**Table 1:** drift characteristics at baseline and during / after sustained roll-tilt.

*Baseline and roll-tilted conditions (**all 29 subjects** included, always median ± one MAD)*					
	**90°LED**	**45°LED**	**upright**	**45°RED**	**90°RED**
Drift amplitude [°/5min]	-3.1 ± 8.8	-1.6 ± 7.6	-0.9 ± 1.3	-0.8 ± 6.4	4.7 ± 8.9
Absolute drift [°/5 min]	6.9 ± 3.5	6.7 ± 2.8	1.6 ± 0.7	6.9 ± 3.1	11.5 ± 3.3
*Baseline and roll-tilted conditions (**n subjects with significant CW drift** included, always median ± one MAD)*					
	**90°LED**	**45°LED**	**upright**	**45°RED**	**90°RED**
	(n=6)	(n=10)	(n=3)	(n=10)	(n=16)
Drift amplitude [°/5min]	7.4 ± 2.1	8.5 ± 2.5	1.9 ± 0.2	9.7 ± 4.3	11.9 ± 3.2
Tc of exp. decay [sec]	224 ± 179	64 ± 49	135 ± 88	212 ± 160	127 ± 103
R^2^-value of exp. decay fit	0.22 ± 0.08	0.23 ± 0.08	0.31 ± 0.01	0.26 ± 0.10	0.29 ± 0.12
*Baseline and roll-tilted conditions (**n subjects with significant CCW drift** included, always median ± one MAD)*					
	**90°LED**	**45°LED**	**upright**	**45°RED**	**90°RED**
	(n=14)	(n=11)	(n=10)	(n=10)	(n=9)
Drift amplitude [°/5min]	-12.3 ± 2.5	-9.2 ± 4.7	-2.1 ± 0.6	-9.2 ± 3.0	-11.6 ± 3.4
Tc of exp. decay [sec]	409 ± 323	113 ± 64	202 ± 136	321 ± 289	55 ± 45
R^2^-value of exp. decay fit	0.49 ± 0.18	0.28 ± 0.16	0.23 ± 0.10	0.35 ± 0.19	0.32 ± 0.09

*Post-tilt conditions (**all 29 subjects** included, always median ± one MAD)*					
	**post 90°LED**	**post 45°LED**		**post 45°RED**	**post 90°RED**
Initial offset re baseline [°]	-1.5 ± 1.8	-2.2 ± 2.3		2.0 ± 1.9	1.1 ± 2.0
Absolute drift [°/5min]	1.9 ± 0.9	2.1 ± 1.2		2.8 ± 1.5	2.6 ± 1.2
*Post-tilt conditions (n subjects with significant drift and initial offset **towards** the previous roll position)*					
	**post 90°LED**	**post 45°LED**		**post 45°RED**	**post 90°RED**
	(n=14)	(n=15)		(n=13)	(n=13)
Initial offset re baseline [°]	-3.0 ± 1.2	-4.2 ± 0.8		3.9 ± 1.5	3.0 ± 1.2
Drift amplitude [°/5min]	2.9 ± 1.2	3.3 ± 1.6		-4.5 ± 1.1	-3.3 ± 1.0
Tc of exp. decay [sec]	67 ± 38	60 ± 25		66 ± 41	87 ± 55
R^2^-value of exp. decay fit	0.39 ± 0.14	0.38 ± 0.14		0.55 ± 0.09	0.50 ± 0.16
*Post-tilt conditions (n subjects with significant drift and initial offset **away from** the previous roll position)*					
	**post 90°LED**	**post 45°LED**		**post 45°RED**	**post 90°RED**
	(n=2)	(n=3)		(n=6)	(n=5)
Initial offset re baseline [°]	4.8 ± 1.3	1.8 ± 1.5		-2.8 ± 1.5	-4.8 ± 1.9
Drift amplitude [°/5min]	-5.5 ± 0.8	-2.3 ± 1.2		2.8 ± 0.2	4.3 ± 1.2
Tc of exp. decay [sec]	16 ± 8	17 ± 7		120 ± 80	198 ± 2
R^2^-value of exp. decay fit	0.28 ± 0.05	0.19 ± 0.01		0.30 ± 0.09	0.64 ± 0.05

**Figure 3:**
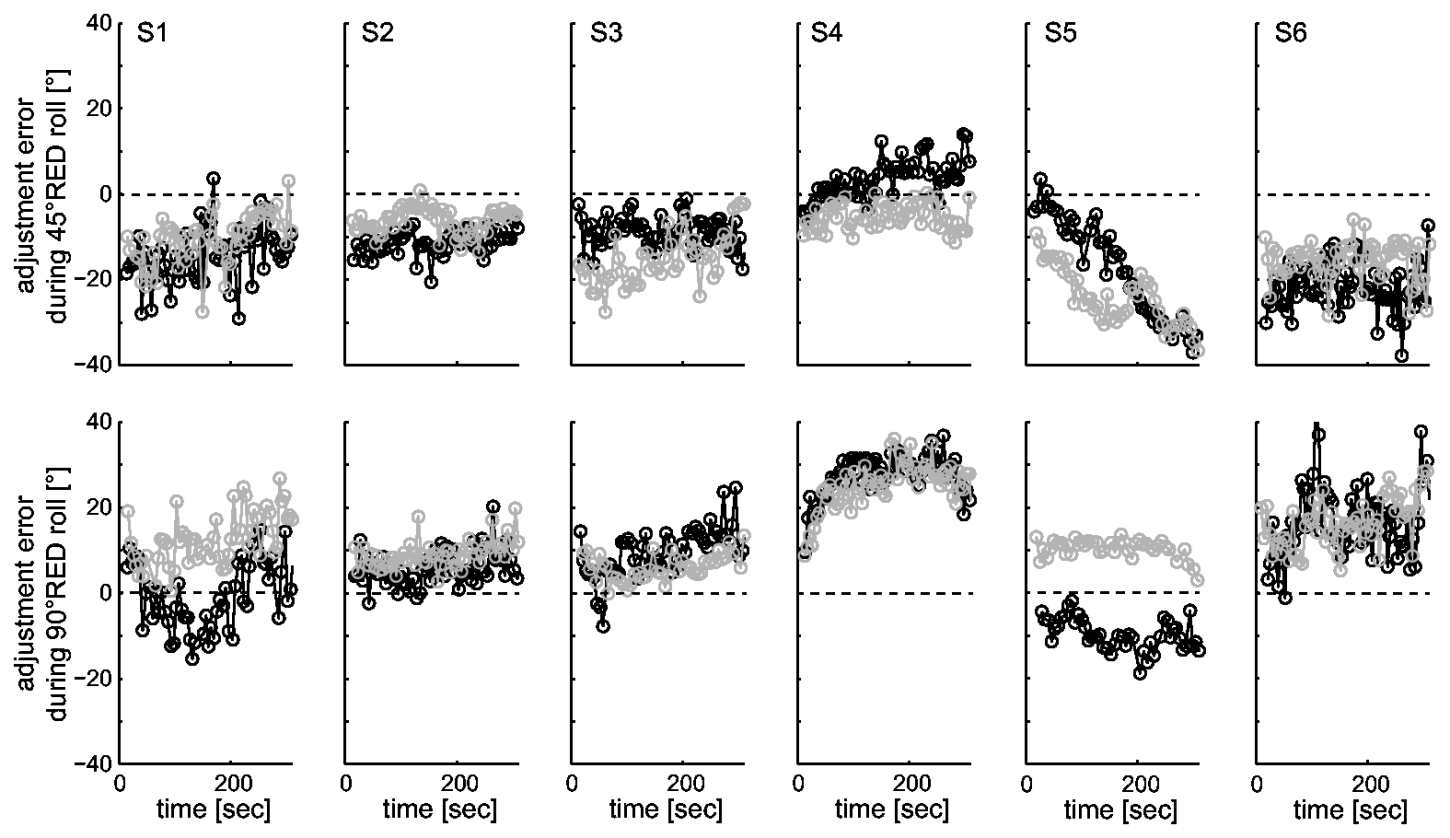
Illustration of drift test-retest reliability in a subgroup of 6 subjects (S1 to S6) for 45° right-ear-down (RED) (top row) and 90°RED (bottom row) roll-tilt. SVV adjustments from the first recording session (black circles) are compared with those from the second session (grey circles) .

In a next step we looked at the amount of SVV drift in individual subjects by calculating the median absolute individual drift amplitudes. This approach revealed absolute median drift of 6.8° (±45° roll) and 9.2° (±90° roll), indicating that at the level of individual subjects drift was indeed occurring (see Table 1). Furthermore, in a subgroup of subjects (n=6), we repeated SVV recordings while roll-tilted (45° and 90° right-ear down (RED)) and increased the duration of roll-tilt to 15 minutes. In these six participants the test re-test reliability during the first five minutes of roll-tilt was high: while at 45°RED four out of six subjects showed a qualitatively similar drift pattern (i.e., same drift direction and similar initial adjustment error), five out of six subjects had comparable drift patterns at 90°RED roll tilt in both sessions (see Figure 3). Overall, both these additional data sets and the findings from a recent study by Tarnutzer and colleagues [43] about the test-re-test reliability of SVV adjustments suggest a stable and individually distinct drift pattern in most subjects. 

Based on these observations we hypothesized that different subgroups exist with distinct drift patterns which may cancel out when pooling all participants. So we calculated the amount and direction of drift in all individual runs and analyzed the drift patterns both with respect to their effect on the size of adjustment errors over time and the direction of drift (CW or CCW). 

#### Linear fit vs. exponential fit

**Figure 4:**
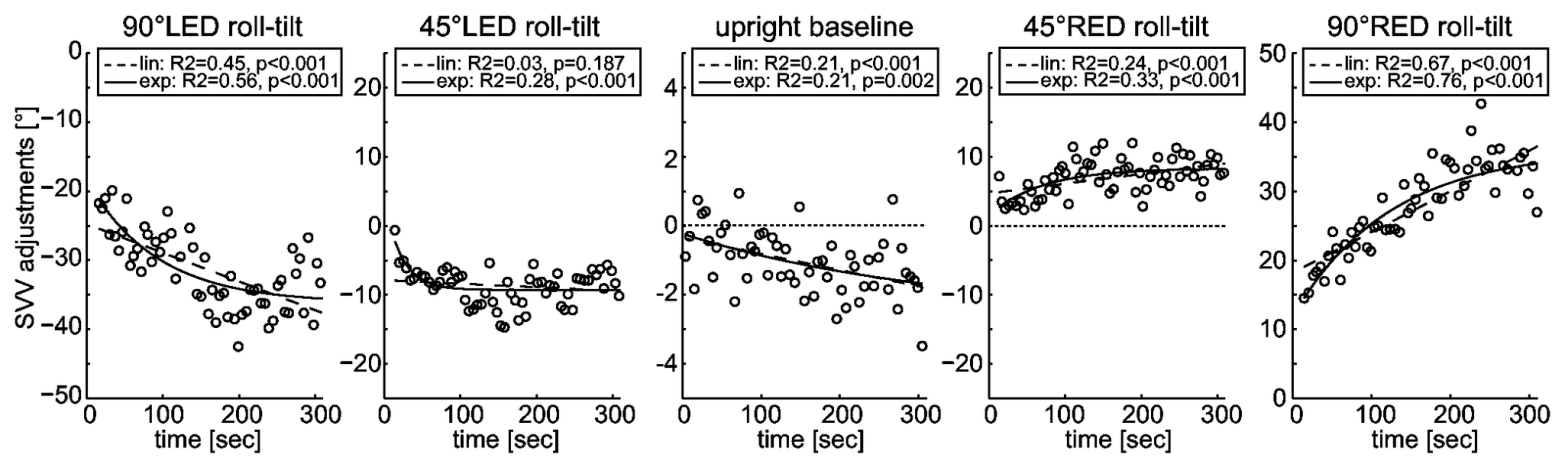
Single subject raw data (with circles referring to individual SVV adjustments) plotted against time for both the baseline (upright) condition and during prolonged roll tilt. Results for both linear (dashed line) and exponential (solid line) fits are provided while corresponding R^2^- and p-values of fitting are shown in insets. This subject had significant drift in all conditions, resulting in an increase of adjustment errors over time both when upright and while roll-tilted. The dashed horizontal lines refer to perfectly accurate (earth-vertical) adjustments. Note that for baseline upright trials the scaling along the y-axis differs from the roll-tilted positions.

**Figure 5:**
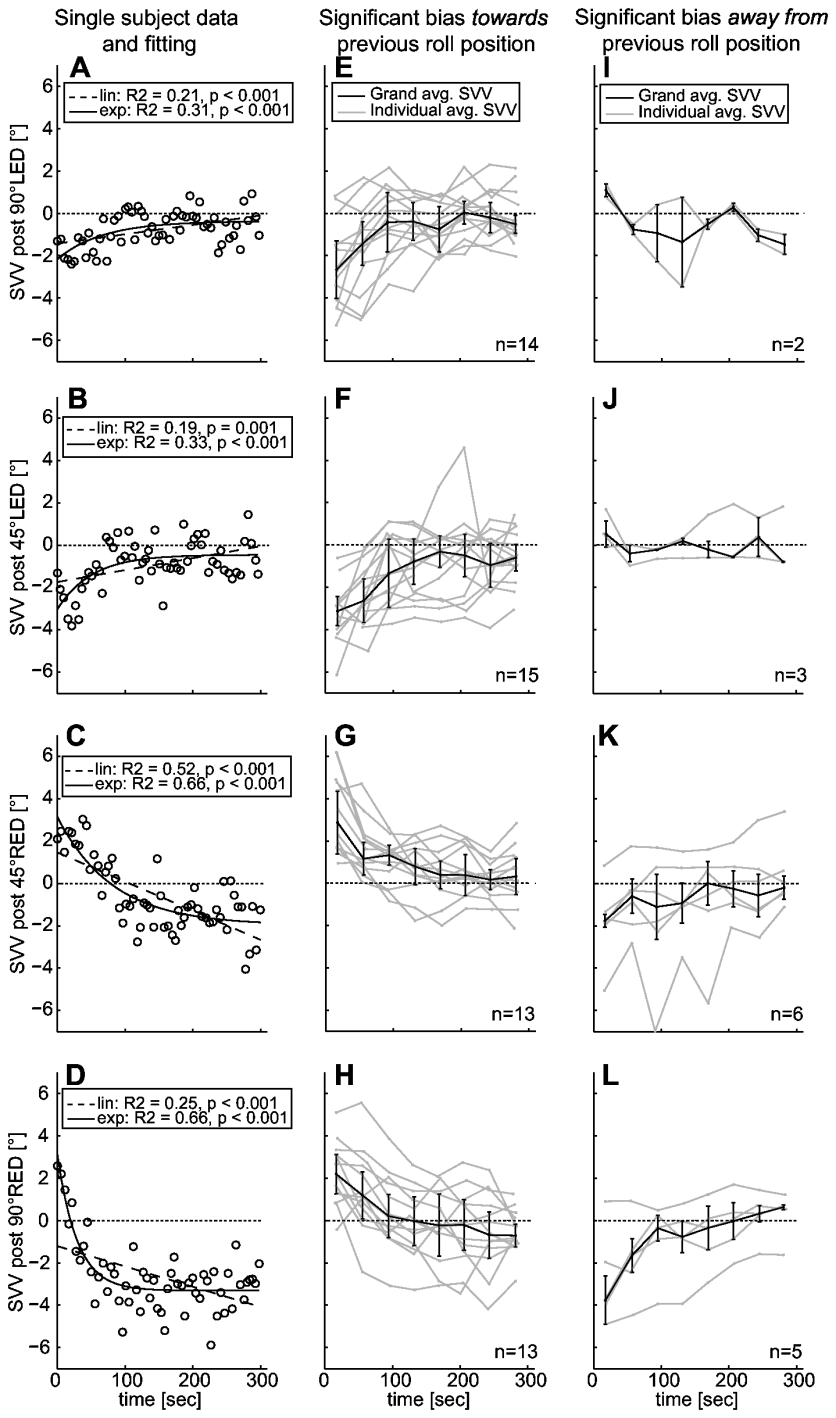
Illustration of perceived vertical upon return to upright position after prolonged roll. The left column (panels A-D) shows both single subject raw data (with circles referring to individual SVV adjustments) and linear (dashed line) and exponential (solid line) fits. R^2^- and p-values of fitting are provided in inlets. Runs [in grey: individual traces; in black: median (± one MAD) traces] with significant exponential decay were further categorized based on the direction of the post-tilt bias: runs that were initially biased towards the previous roll orientation are shown in the middle column (panels E-H), runs that were biased away from the previous roll orientation are presented in the right column (panels I-L), always indicating the number of traces (n) that met the criteria. To improve the illustration of drifting traces in the middle and right column, single adjustments were assigned to one of ten bins of equal length and the individual median was calculated. However, the fit was obtained from the raw data. The dashed horizontal lines refer to perfectly accurate (earth-vertical) adjustments.

 Both the linear function (Eq. 1) and the exponential function (Eq. 2) were fit to all individual data sets, as illustrated in Figure 4 (baseline upright and during prolonged roll-tilt) and further below in Figure 5 (post-tilt conditions) for a single subject. Goodness of fit (median R^2^-value ± one MAD) was significantly better (Friedman’s test) when using the exponential fit compared to the linear fit at baseline before roll-tilt (p=0.041, 0.10 ± 0.09 vs. 0.08 ± 0.07, exponential vs. linear fit), during prolonged roll-tilt (p=0.026, 0.21 ± 0.14 vs. 0.15 ± 0.13; all roll-tilted conditions pooled) and immediately after returning back upright (p<0.001, 0.19 ± 0.16 vs. 0.13 ± 0.12; all post-tilt conditions pooled). Based on these findings we opted for using the exponential decay function for further drift analysis of all traces. Arguably, this result in favor of the exponential fit may be related to the fact that the exponential fit has an additional (3^rd^) parameter compared to the linear fit. 

#### Drift at baseline recordings

**Figure 6:**
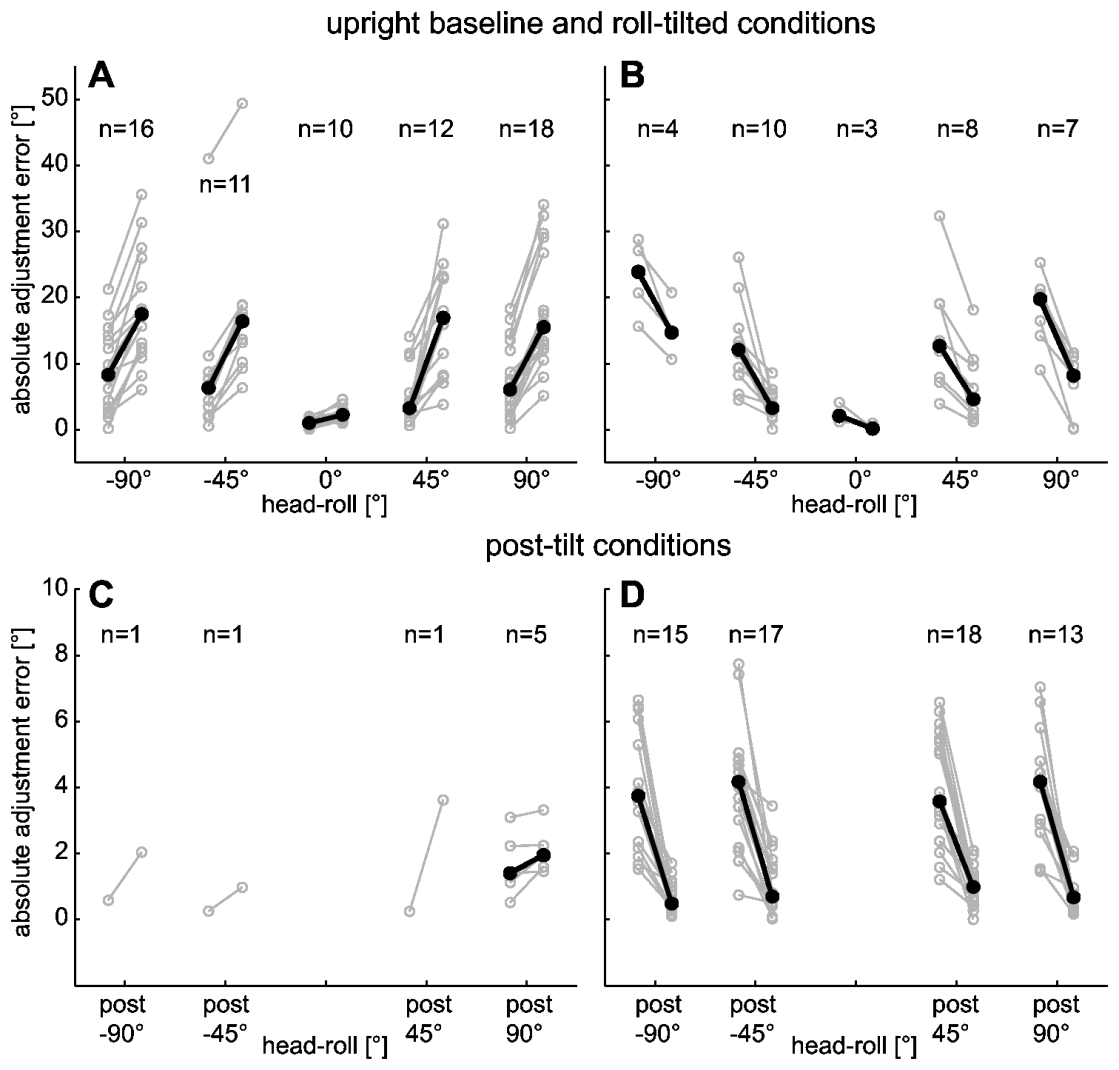
Individual (in grey) and overall (in black) median adjustment errors at the beginning (left column) and at the end (right column) of each five-minute run derived from the fitted traces, for both upright baseline and roll-tilted conditions (panels A and B) and post-tilt conditions (panels C and D). Results for the different head-roll orientations are shown separately. The number of traces (n) that met the inclusion criteria (i.e., significant drift over the 5-minute recording period) is shown above the data. While panels A and C show all traces with significantly increasing errors over time, panels B and D illustrate traces that had a significant decrease in error.

During baseline recordings in upright position, 13 out of 29 subjects had significant exponential drift, either CW (n=3) or CCW (n=10). In the majority of subjects with significant drift (10/13), SVV accuracy (comparing the first vs. the last value of the fitted function) over the five-minute recording period decreased, while only 3/13 subjects showed improved SVV accuracy (see Figure 6A, B).

#### Drifts during prolonged roll-tilts

**Figure 7:**
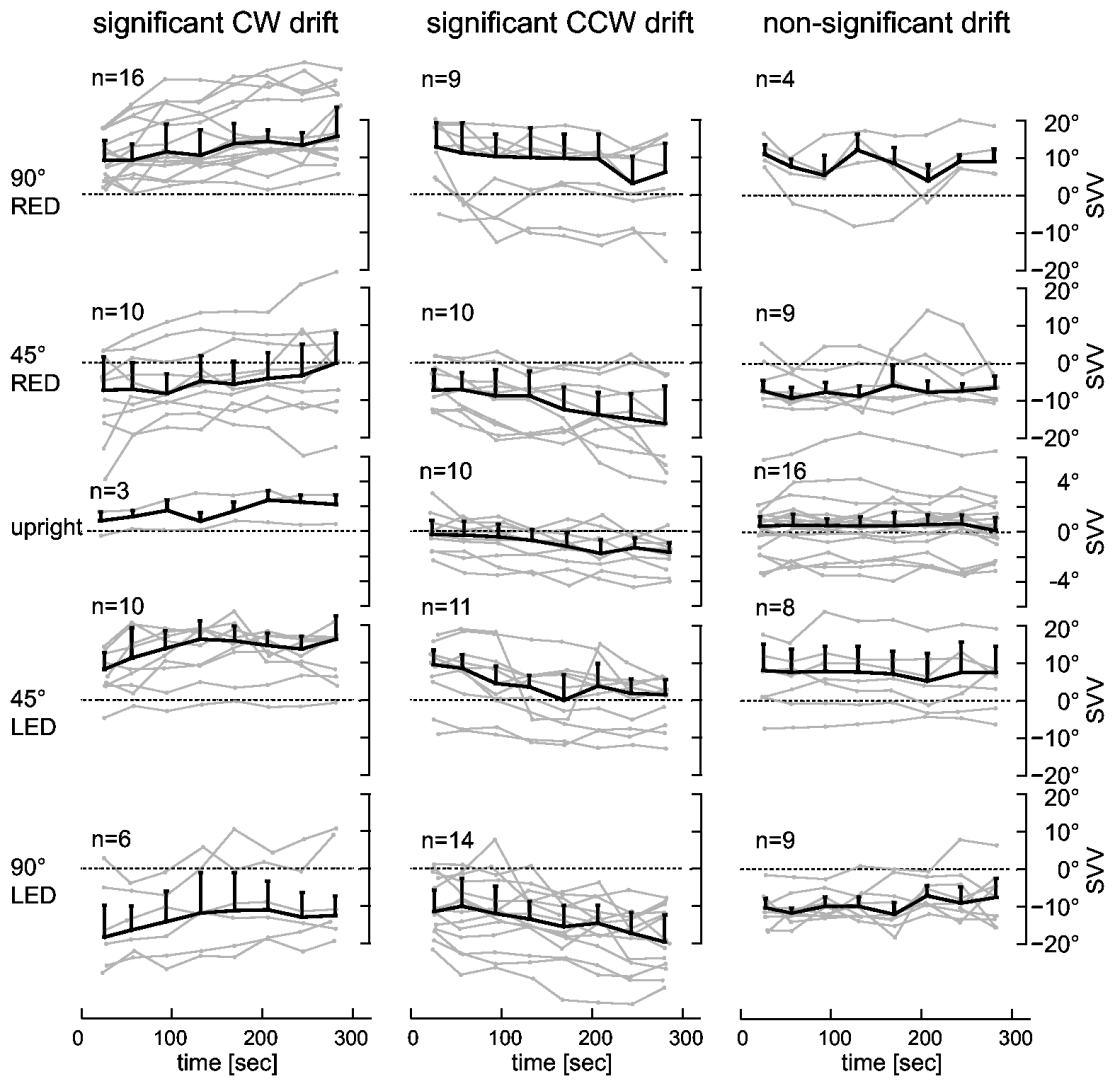
Individual (in grey) and median (in black) adjustment errors while roll-tilted are plotted against time. Traces with significant (p < 0.05) CW, significant CCW and non-significant drift while roll-tilted and in upright position are shown in separate columns, always indicating the number of subjects (n) that met the criteria for a given trial type. Note that for baseline upright trials the scaling along the y-axis differs from the roll-tilted positions. For a more detailed description see figure legend of Figure 2.

Significant exponential drift was found in 86 of 116 traces (for detailed numbers on drift amplitude and goodness-of-fit see table 1). The median time constant (Tc) of decay in these 86 traces was 121sec (± 101 sec, ± oneMAD). Median drift amplitudes (over the five-minute recording periods) for all roll-tilted conditions ranged between 7.4 and 11.9° (for CW drifts) and between -9.2 and -12.3° (for CCW drifts). For the different roll-tilted positions (±45°, ±90°) measured drift amplitudes were not significantly different (Friedman’s test, p >0.05). Based on the drift pattern, individual runs were separated into three different categories (significant CW drift, significant CCW drift, non-significant drift). Drift was significant in 20 to 25 (out of 29) subjects in the different roll-tilted conditions as shown in Figure 7. 

We further analyzed individual runs with significant drift with regards to their impact on the accuracy of SVV adjustments (defined as the change in errors relative to earth-vertical over the 5-minute period). Depending on the drift direction and the starting roll position, accuracy of estimates may improve or deteriorate. In Figure 6 changes in SVV derived from the fitting parameters obtained by exponential decay analysis are shown, distinguishing between runs with significant *increase* (panel A) and *decrease* (panel B) of errors relative to true earth-vertical over time. While significant increases and decreases of errors were found in about the same number (roughly one third) of subjects (8 to 12 out of 29) for ±45° roll, errors increased significantly in 60% (16 to 18 out of 29) for ±90° roll orientations, while significant decreases were noted only in about 20% (see Figure 6, panels A and B, for exact numbers). 

**Figure 8:**
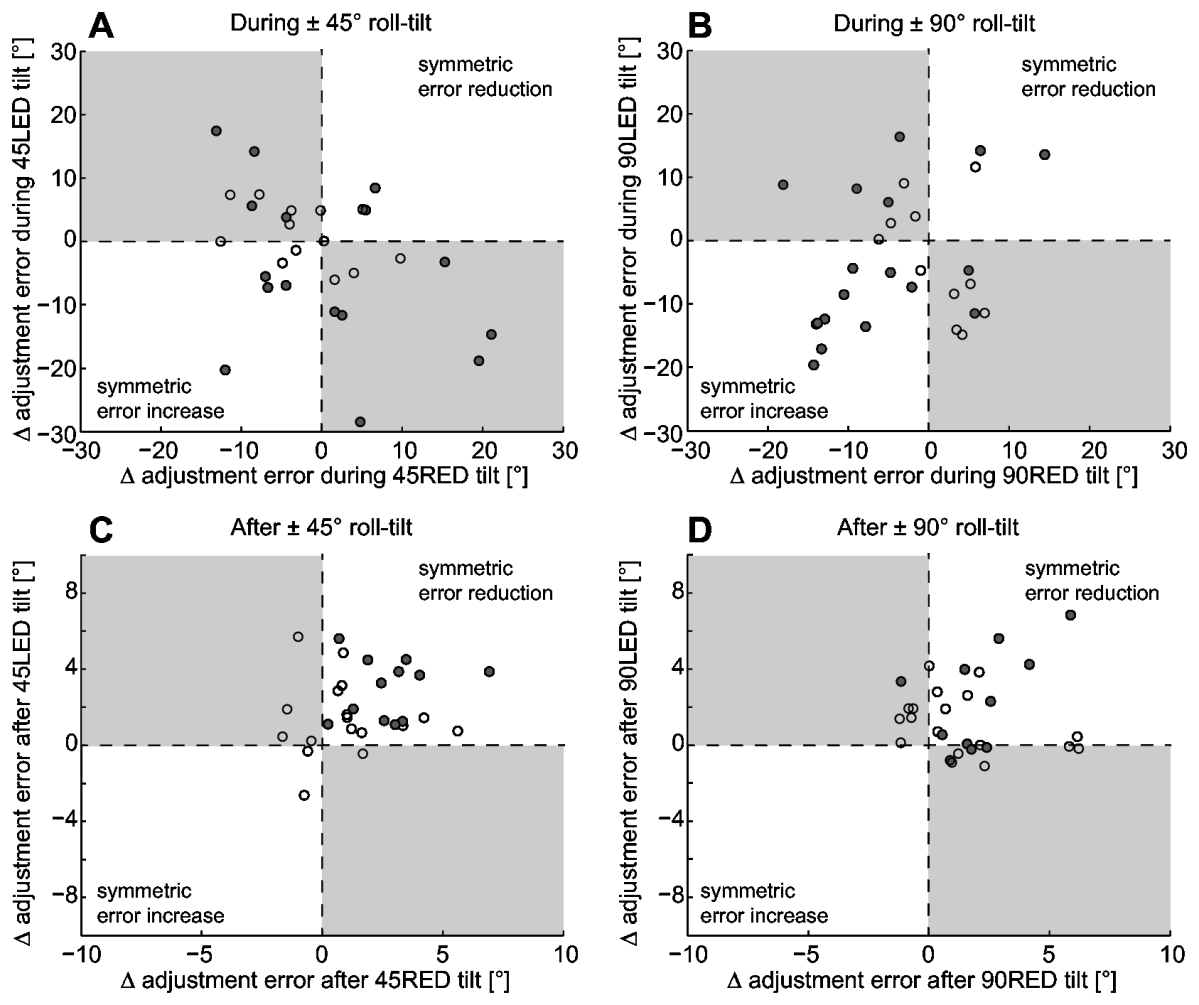
Comparison of changes in adjustment errors over the 5-minute recording periods during (panels A and B) and immediately after (panels C and D) prolonged roll-tilt (±45° or ±90°). Grey filled circles refer to runs with significant drift for both RED and LED while empty circles indicate runs with non-significant drift in at least one of the two conditions. Each panel is split up in 4 areas separated by dashed horizontal and vertical lines along zero: while the grey-shaded areas indicate runs where drift was not symmetric (e.g. error was increasing at 45° RED and decreasing at 45°LED or vice versa), trials with symmetric drift for LED and RED (for a given roll angle) will fall in the white areas, either in the lower left corner (if adjustment errors increased over time) or in the upper right corner (if adjustment errors decreased over time).

On an individual subject basis we further analyzed drift patterns for symmetry. We therefore compared individual drift responses leading to either significant error increases or decreases over time for RED vs. LED. While at ±45°, significant drift was found in 16 subjects for both LED and RED position, 18 subjects presented with significant drift both at +90° roll-tilt and -90° roll-tilt. For conditions with prolonged static roll at ±45° we noted significant error reduction (2/16) or significant error increase (4/16) for both RED and LED in 38% or 6 out of 16 runs. For conditions with ±90° roll-tilt, significant error reduction (2/18) or significant error increase (10/18) for both RED and LED orientation was found in 67% or 12 out of 18 runs. These results are summarized in Figure 8 (panels A and B). In three out of 116 runs we found (based on visual inspection) drift to change direction, consistent with oscillations, however, in all remaining runs drift was either ongoing (without a change in direction) or had stopped by the end of the 5-minute recording period. 

We hypothesized that the adjustment errors at the beginning of the 5-minute recording period may affect the direction of drift, i.e. being consistent with a tendency to drift either towards the true earth-vertical or towards the body-longitudinal axis. However, for roll-tilted conditions (pooling ±45° and ±90° roll angles) no such correlation was found between the initial adjustment errors and the drift amplitude over the five-minute recording period (R^2^ = 0.28, slope = -0.83, 95% CI = -0.99 to 0.80).

 The drifts observed here do not only affect the accuracy of perceived vertical but will also modulate its precision. For our data analysis, we did not remove drift in the roll-tilted positions. However, to quantify effects of drift on the precision of SVV estimates while roll-tilted, we removed the drift from all roll-tilted traces for this additional data analysis. We then compared the trial-to-trial variability (corresponding to one MAD) in the original and the modified data sets. Both sets showed a roll-angle dependent increase in trial-to-trial variability (see Table 2). However, the decrease in trial-to-trial variability noted when removing individual drift was significant (p<0.05, Friedman’s test) for two (90RED and 45LED) of the four roll-tilted positions only. 

**Table 2:** trial-to-trial variability.

*Baseline and roll-tilted conditions (always median ± one MAD)*					
	**90°LED**	**45°LED**	**upright**	**45°RED**	**90°RED**
Drift removed [°]	2.5 ± 0.5	2.0 ± 0.6	0.6 ± 0.2	2.2 ± 0.7	2.1 ±0.5
Including drift [°]	3.1 ± 0.9	2.5 ± 0.8	0.6 ± 0.2	3.1 ± 1.0	2.9 ± 0.8
Drift removed vs. including drift	p>0.05	p<0.05*	p>0.05	p>0.05	p<0.05*

#### Drifts in the post-tilt conditions

Individual post-tilt traces were initially fit using both the linear and the exponential function, as shown in a typical subject in Figure 5, left column. The goodness of fit was significantly better for the post-tilt conditions when using the exponential fit compared to the linear fit.

**Figure 9:**
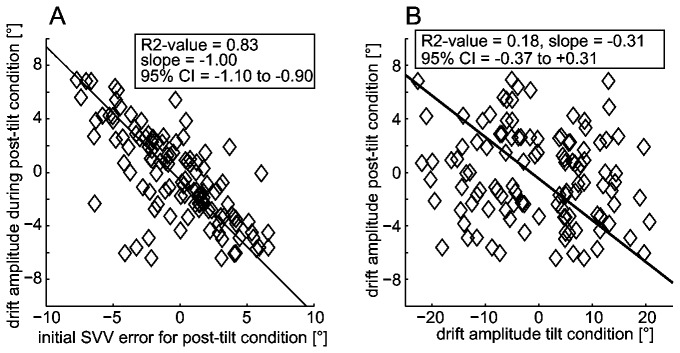
Characteristics of the post-tilt drift amplitude: correlation with initial offset and drift during prolonged roll-tilt. Panel A: Correlation analysis between the post-tilt drift amplitude and the initial post-tilt bias when returning to upright position using principal components analysis (PCA). The diamonds refer to single runs, the solid line indicates the fit obtained. In an inset, goodness of fit (R^2^-value), the slope and the 95% CI of the slope are provided. Panel B: Comparison of the individual drift amplitudes during prolonged roll-tilt and immediately after returning back upright using principal components analysis (PCA). The diamonds refer to single runs, the solid line indicates the fit obtained. In an inset, goodness of fit (R^2^-value), the slope and the 95% CI of the slope are provided.

Depending on the different roll-tilt angles, exponential drift was significant in 17 or 18 out of 29 subjects in the post-tilted conditions. Pooling all post-tilt conditions, significant decreases in adjustment errors were found in 54% (63/116) of trials, while significant increases of adjustment errors occurred only in 7% (8/116) of runs (see Figure 6, panels C and D for exact numbers). Errors at the beginning of the post-tilt period were a precise predictor of the post-tilt drift amplitude, as shown by the highly significant correlation (Figure 9, panel A) between these two parameters (R^2^ = 0.83, slope = -1.00, 95% CI = -1.10 to -0.90), indicating that subjects successfully restored the original pre-tilt percept of vertical within 5 minutes after returning to upright position. Noteworthy, most subjects reported a sensation of being roll-tilted to the side opposite to the previous roll. This sensation usually diminished within a few minutes.

Runs with a significant post-tilt drift towards baseline initially deviated significantly (p < 0.001, Fisher’s exact test [46]) more frequently towards the side of previous roll (47%, 55/116 runs) than into the opposite direction (14%, 16/116 runs). The initial bias was of similar size for all post-tilt conditions with median values ranging between ±3.0 and ±4.2° (see Table 1). In five subjects such a bias towards the direction of previous roll followed by significant exponential decay was found in all four post-tilt conditions and in another five subjects this was the case in 3 out of 4 post-tilt conditions. As for the runs during prolonged roll-tilt, we analyzed individual drift patterns of the post-tilt traces for symmetry. Symmetry would support the hypothesis that adaptation of a prior contributes to the drift and subsequently to the post-tilt bias. Comparing individual drift responses leading to either significant error increase or decrease over time for RED vs. LED, we noted a symmetric reduction in adjustment errors of 100% (12/12; ±45° roll tilt) and 64% (7/11, ±90° roll tilt) in RED and LED conditions, respectively (Figure 8, panels C and D).

Traces that demonstrated a post-tilt bias towards the previous roll orientation and significant decrease in errors over time (see Figure 5) were further analyzed. The overall median (± one MAD) time constant of exponential decay was 71 (± 36) seconds. Noteworthy, decay dynamics were similar for all four post-tilt conditions, showing no significant differences (p>0.05, Kruskal-Wallis non-parametric ANOVA) in decay time (see Table 1 for details). 

We hypothesized that the drift observed during and after prolonged roll-tilt originate from a common source. For example, adaptation of peripheral sensors may cause such a behavior. If indeed such a common mechanism were contributing to the observed drift, patterns of drift during and immediately after roll-tilt should show common features on an individual level, i.e. they should correlate. We therefore compared the individual drift amplitudes (and directions) during and immediately after prolonged roll-tilt. When comparing repetitive SVV adjustments on an individual subject basis, we found no significant (R^2^ = 0.18, slope = -0.38, 95% CI = -0.37 to 0.31) correlation between the drift amplitudes during and immediately after roll-tilt (Figure 9, panel B), suggesting that the post-tilt bias is not related to the drift during the preceding roll-tilt position. These findings speak against a common underlying (peripheral) source for drift in these situations. 

#### Spectral density analysis and autocorrelation of drift

Previously, serial SVV adjustments were found to be dependent [17]. Drift in SVV may potentially be related to the self-similarity of consecutive adjustments as reviewed by Torre and Wagenmakers [45]. We therefore calculated the log-log power spectrum for repetitive SVV adjustments for all subjects and conditions. Overall we found linear decay of the log-log power-spectrum with slope *β* of varying size. For the baseline recordings in upright position, the median (± one MAD) slope of *β* was -0.42 (± 0.27), while for roll-tilted positions median slopes ranged between -0.86 and -0.93, and for post-tilt conditions between -0.64 and -0.69, indicating that serial correlations were less robust (for an 1/*f* ^*β*^ process slope *β* typically ranges between -0.5 and -1.5) in the baseline recordings in upright position than in roll-tilted conditions and in post-tilt conditions. Furthermore, the slope was significantly larger for roll-tilted conditions compared to the post-tilt conditions (Friedman’s test, p < 0.003). Auto-correlation analysis for each condition obtained separately showed a strong correlation only for up to 2 to 3 trials, whereas afterwards a gradual decay over the next 10 to 15 trials was found, reaching zero within approximately 15 trials. 

### Subjective haptic vertical paradigm

Theoretically, the drift during prolonged roll-tilt could be related to retinal input and the torsional position of the eyes. In order to identify vision-related effects on the stability of the internal estimate of direction of gravity, a second session using a non-visual task was obtained in six subjects. The median number of haptic alignments obtained within the 5-minute periods in this haptic paradigm was 55 trials (± 4; one MAD). As for the analysis of the SVV data we will apply the exponential fitting algorithm described above (Eq. 2).

#### Drift at baseline recordings

At the beginning of the baseline recording period, adjustments deviated CCW in 5 out of 6 subjects. During the five-minute block, absolute drift amplitudes averaged at 3.1 ± 2.1° (median ± oneMAD). In three subjects, drift was statistically significant (being clockwise in all of them and resulting in a reduction of adjustment errors over time).

#### Drifts during prolonged roll-tilts

**Figure 10:**
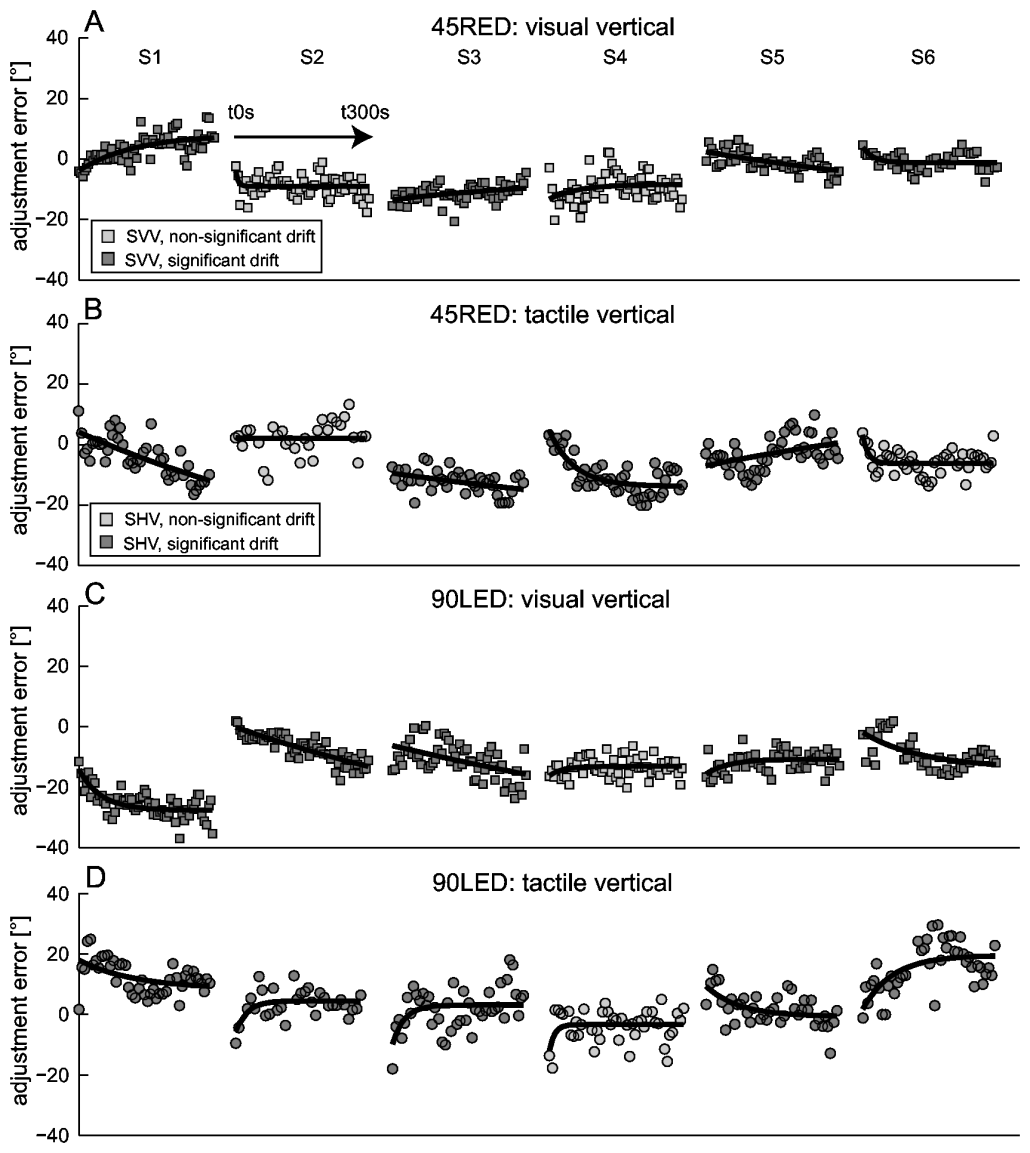
Comparison of individual adjustment errors in the SVV paradigm (squares) and the SHV paradigm (circles) during prolonged roll-tilt for two of the four roll-tilt conditions studied (panels A and B: 45RED; panels C and D: 90LED) in those six subjects (S1-S6) that completed both paradigms. All runs are plotted against time starting at time t0 seconds (t0s) and ending at time t300s (as shown in the inlet in panel A). Runs with significant exponential drift are presented with dark grey symbols, while those runs with non-significant exponential drift are shown in light grey. The solid black lines indicate the fit of the exponential decay function (Eq. 2).

From the 24 runs obtained during prolonged roll-tilt, significant drift could be found in 15 (63%), with median absolute drift amplitudes ranging between 6.4 to 14.4° (see Figure 10). Error increases over time were noted in 6 runs (90LED and 45LED 1 each, 45RED and 90RED 2 each), while in the remaining 9 runs with significant drift adjustment errors decreased over time (90LED: 4, 45LED: 2, 45RED: 1, 90RED: 2). Median Tc of drift (using the exponential fit) for the four roll-tilted positions studied ranged between 66 and 879 seconds. 

### Comparison of SVV and SHV adjustments

A subject-by-subject comparison of the individual drift characteristics across different modalities was obtained in those six subjects that performed both the SVV and the SHV task during prolonged roll-tilt. Significant drift in both paradigms was found in 13 out of 24 runs, with drift going into opposite directions (CW vs. CCW) in the majority of these runs (9/13). While an error increase was observed in the majority of runs with significant drift (18/24) for the SVV, error reductions were more frequent than error increases (10 vs. 7) in the haptic vertical paradigm. Taken together, subjects that showed significant drift in the SVV paradigm also tended to do so in the SHV paradigm in about 50% of cases, with drift going into opposite directions in these paradigms in a majority of cases (69%). 

## Discussion

Our results show a significant difference in the SVV drift behavior at the group level and at the level of individual subjects. While for the entire group of participants drifts during prolonged roll-tilt were minor and non-significant, individual subjects indeed showed significant drifts of perceived vertical. These findings indicate that the direction and amplitude of drift is individually distinct and roll-angle-dependent. Test-re-test reliability over time as tested in a subgroup of subjects was found to be high, suggesting that individual drift patterns remain stable over time. As a consequence of drift, individual SVV accuracy increased or decreased, depending on the adjustment error at the beginning of the five-minute period and the direction of drift. Immediately upon return to upright position perceived vertical was biased towards the previous roll orientation - followed by exponential decay - in half of the runs. In the subgroup of subjects that performed the haptic vertical task- being devoid of visual input – we also observed significant drift typically pointing in the opposite direction than drift in the SVV condition during prolonged roll-tilt. 

Adaptation is a mechanism observed in virtually all sensory systems [32,47] and explanations of its occurrence have focused on improvement of discriminability around the adapter [48]. For example, previous studies reported systematic biases in the estimation of orientation, contrast and direction of subsequent stimuli after prolonged exposure to a visual stimulus of a particular orientation [49–51]. Similar observations were made for prolonged vestibular stimuli as constant-velocity rotation or caloric irrigation: after the original nystagmus stops, a reversal of nystagmus with slow phases in the opposite direction develops [52,53], likely reflecting an adaptive mechanism [54], either related to habituation of the peripheral sensors themselves or of the central nervous system. We will discuss our findings and possible explanations in this context. 

### Possible explanations for the drifts observed during prolonged roll-tilt

Over the five-minute recording periods, the majority of subjects with significant SVV drift continued to drift or was stable, and only a few changed direction of drift. Response adaptation to ongoing static roll-tilt or constant velocity centrifugation was previously reported both at the level of the otolith afferents [8,55,56] and the vestibular nuclei [57,58]. Similarly, the proprioceptors responsible for touch and pressure showed adaptation with constant pressure [59,60], which has led some authors to favor somatosensory adaptation as underlying cause of SVV drift [14]. Based on simulations of perceptual effects of adaptation to visual motion direction and contrast, Series and colleagues have proposed that the cortical areas that decode the neuronal activity to a perceptual estimate are unaware of the adaptation-induced neural changes in these paradigms [47]. In analogy, prolonged roll-tilt leading to adaptation of the peripheral sensors and consecutively to a change in the afferent firing rate could then be erroneously interpreted by the brain as a change in the subject’s roll orientation. 

#### Adaptation of the peripheral sensors

Peripheral sensory adaptation typically leads to a change in the current firing rate towards the previous (baseline) firing rate [8,32], i.e. the difference of the firing rate between the actual, roll-tilted position and the previous, upright position decreases. This change in the firing rate might be interpreted by the brain as a shift of the current position towards a smaller roll-tilt angle. Therefore, when adjusting the luminous arrow along perceived vertical, the brain will compensate for a smaller fraction of body roll angle than at the beginning of the roll-tilt period. An initially observed A-effect is predicted to increase, while an initial E-effect is expected to decrease during prolonged roll-tilts. The SVV drifts in our subjects only partially followed this pattern. Noteworthy, about one third of subjects each had their adjustment errors increase, decrease or remain stable over time for ±45° roll positions.

#### Noise from the semi-circular canals

Sensory input from the otolith organs and the semicircular canals (SCCs) are combined within the brainstem vestibular nuclei. This vestibular convergence predicts that sensory noise from the SCCs also affects the otolith-derived estimate, therefore influencing paradigms in static roll-tilted positions without any SCC stimulation. During and after constant velocity rotations in the yaw plane for one to several minutes using a centrifuge or a gondola [38,61,62] and after constant velocity rotations in the roll plane [15], studies have shown a prolonged effect of semicircular canal stimulation on perceptual estimates of direction of gravity, decaying over 30 to 45 seconds after cessation of rotation. Theoretically, this decay could be reflected in drift of the perceived direction of gravity. However, similar data is not available for brief velocity steps as used here. It therefore remains unclear, whether a brief velocity step shifting whole-body roll orientation by 45 or 90° also causes a memory effect of SCC stimulation that affects subsequent SVV adjustments. Turntable-repositioning maneuvers must be performed with accelerations below the threshold of SCC stimulation to rule out a memory effect of SCC stimulation. Theoretically, integration of the canal signal may be used to determine head roll position. However, previous research has indicated that the brain is relatively poor in using intergrated canal signals for updating changes in position. Using single axis rotations (including roll while upright, which stimulates the otoliths and canals, and supine, when only the canals are stimulated), Klier and colleagues reported perceptual localization performance being low when only canal signals were available [63].

#### Random walk processes

Sensory signals such as the SCCs’ resting discharge firing rate, the macular vestibular afferents and from the proprioceptors all contain noise, which for estimating the direction of gravity leads to variability and potentially to random walk processes. A random walk (i.e., as defined by Merriam Webster ‘a process (...) consisting of a sequence of steps (...) each of whose characteristics (as magnitude and direction) is determined by chance’) with low-frequency oscillations may result in transient drift and could therefore at least partially explain the individual SVV drift patterns. Theoretically, a combination of several mechanisms, including some kind of adaptation (peripheral or central), which would uniformly trend into one direction for all subjects in a given roll-tilt position, and a random-walk component, whose direction of drift would be random across subjects, could explain the drift during prolonged roll-tilt. Variation in the relative size of drift due to adaptation and random walk could then explain the heterogeneity of drift patterns found amongst the subjects. By definition a random walk will not be stable over time, i.e., its position is determined by chance. To further evaluate the role of random walk processes, we repeated SVV measurements in six subjects. Both our findings from this subgroup (showing a qualitatively similar drift pattern in 75% of cases) and a recent study by one of the authors reporting individually distinct, but direction-specific drift over the duration of at least one hour [43] suggest that drift in SVV is a fairly stable pattern over time in individual subjects. These observations put a relevant contribution of random walk processes to SVV drift at the level of individual subjects into question, but do leave the possibility for random walk effects at the group level. 

#### Direct motor effects and serial correlations

Theoretically, direct motor effects could also contribute to drift patterns. However, the kind of motor task required here (turning a wheel with two fingers and pushing a knob with another finger) is rather simple and requires few degrees of freedom. We have previously demonstrated that under optimal conditions (i.e. with a structured and illuminated background and when providing a visual reference along earth-vertical) this task can be completed with an at least twofold higher precision than the SVV task in darkness [17]. This makes a relevant contribution of direct motor effects unlikely. Furthermore, based on the autocorrelation analysis and the spectral density analysis performed on all SVV traces, serial correlations seem to contribute to the drift only on the short term, as correlations were lost within 10-15 trials. In this analysis, we did not find evidence for random serial correlations. 

#### Central adaptation

Based on the considerations presented above, we propose that a single mechanism, such as random-walk effects, peripheral adaptation and direct motor effects – although possibly contributing to SVV drift - cannot sufficiently explain the drift patterns observed. At the same time central adaptation mechanisms likely add to the individually distinct drift. Shifting the peak of the distribution of prior knowledge or changing the width of the prior might be such potential central mechanisms. With the prior probability distribution being narrow, more weight is put on prior knowledge when determining the posterior probability distribution during prolonged roll-tilt; shifting its peak towards the body roll-position. Likewise, broadening the prior probability distribution will result in weighting more the sensory input when calculating the posterior during roll-tilt, shifting its peak away from the body roll-position. As pointed out by MacNeilage and colleagues [31], priors have large variance, contributing little to the posterior probability distribution if rich sensory input is available. With increasing roll, however, sensory input becomes noisier, as has been reported for otolithic input [5,39]. As a result, the variability in drift direction observed between individual subjects could result from varying noise levels on the sensory signal or individually distinct strategies in how to weight prior knowledge and sensory input when roll.

### Drift of SVV during Prolonged Roll-Tilt and Ocular Torsion – Is There a Link?

Taken together, we cannot attribute the drift in SVV adjustments during prolonged roll-tilt to a single process. Likely, a combination of different mechanisms such as a shifting prior probability distribution, peripheral sensory adaptation and random walk processes is responsible for the drifts and their variability within and between individual subjects. How could future experiments help identifying other relevant peripheral or central mechanisms of adaptation to explain the drift pattern during prolonged roll tilt? While there is sound evidence for peripheral adaptation during prolonged roll, the central contribution remains elusive. First the cortical and cerebellar areas responsible for such presumed central adaptation need to be identified. One potential location are the temporo-parietal cortical areas involved in integrating multisensory input to generate the percept of vertical [1]. Studying drift patterns in patients with lesions in these cortical areas could help answering the question to which extent and in what way drift in perceived vertical during prolonged roll is modulated by central adaptation. 

While the SVV allows a fast, easy-to-understand and reproducible assessment of perceived direction of gravity, it also has restrictions and confounding factors. Probably the most important potential confounder is the fact that head roll provokes counter-rolling of the eyes (ocular counter-roll or OCR). The gain of compensatory OCR (i.e. the fraction of compensatory ocular torsion relative to head roll) ranges between 0.05 and 0.25 for passive head roll and shows sinusoidal modulation with roll angle [64]. Likely, OCR affects SVV adjustments. Wade and Curthoys have proposed that the brain is unaware of OCR and that this is the basis for the E-effect at small roll angles up to 40° [22]. Thereby OCR may contribute to errors in SVV when roll-tilted; however, more important for the current study is the question, how stable ocular torsion (OT) over time is and how this affects the SVV when the subject is roll-tilted.

Human data on the stability of torsional eye position during prolonged roll-tilt is scarce. Keeping healthy human subjects (n=2) in a static roll-tilted position (±60°) during 10 minutes, Diamond and colleagues found variations of torsional eye position in the range of 2.5° to 4° [35]. Noteworthy, only one of the two subjects showed increases (+60° roll-tilt) or decreases (-60° roll-tilt) in torsional amplitude over the 10-minute period, while in the other subject OCR remained stable. As possible causes of such variations in OCR during static roll-tilt these authors discussed several mechanisms, including spontaneous changes in extraocular muscle tone, a failure of utricular and saccular nerve fibers to supply a stable tonic discharge and other CNS influences. Noteworthy, all these mechanisms would add noise to torsional eye position. More recently, in a study by Pansell and colleagues OCR was elicited by 30° head-on-trunk roll [9]. When keeping the head in this roll-tilted position over 10 minutes, the amount of OCR decreased linearly during this period. In a second study the same group reported OCR increases (n=2) or decreases (n=9) over time during 30° of head roll RED [65]. Based on these reports, drift in SVV could be considered as being secondary to drift in torsional eye position. Such a mechanism would be based on the previously proposed hypothesis that the brain is unaware of OCR [22]. As a consequence, drift in OCR would not be compensated for by the SVV, resulting in drift of the perceived vertical when assessed by a vision-dependent paradigm. Based on the data published by Pansell [9,65], we calculated an average reduction of OCR in the range of approximately 1-2° over 10 minutes of static roll-tilt at 30°. However, even when assuming a perfect correlation between OT and SVV, we estimate that drifting OT might explain only about 10-20% of SVV drift. With regards to the amplitude and direction of drift of OT, data is available only for 30° head-on-trunk roll [9]. Under the assumption that the direction of drift is the same for larger roll angles (i.e. OT decreases over time, SVV errors are predicted to decrease over time in the case of an initial E-effect and to increase in the case of an initial A-effect. Overall, such a pattern was found only in a part of all subjects that drifted.

Taken together, variation in OCR may contribute to the SVV drifts observed here. However, to which extent OT drift impairs the stability of perceived vertical cannot be determined from the SVV paradigm applied here. To further assess the role of visual input we therefore repeated the paradigm in a subgroup (n=6) using a haptic task devoid of any visual input instead. The main finding from this control experiment – demonstrating significant drift in 63% of runs – is that shifts in perceived direction of gravity over time are a more global phenomenon, not being restricted to the SVV but also occurring independently from any retinal input. Drift in torsional eye position – as proposed further above as potential source of SVV drift - falls short to explain the drift noted in the SHV paradigm. 

### Comparison of SVV and SHV Data during Prolonged Roll-Tilt

The six subjects that completed both the SVV and the SHV paradigm showed an oppositely directed drift behavior in the majority of runs with significant drift during prolonged roll-tilt. This observation may suggest that the direction of drift does not only depend on the selected roll-tilted position, but is also paradigm-dependent. While both the SVV and the SHV can be used to indicate perceived direction of gravity, relevant differences in adjustment performance need to be considered. First, no systematic roll-angle dependent over- and under-compensation of body tilt are found in the SHV [24]. Second, the SHV is often biased CCW relative to earth-vertical by several degrees when performed with the right hand. Both differences in the sensory input available in the two paradigms and how it is centrally processed may contribute to the discrepancies in perceived vertical as indicated by these two paradigms. In analogy, drift on a sensory input signal may be processed differently, potentially resulting in distinct drift patterns in the same subject for the SVV and the SHV. For example, possible adaptation of felt hand position could influence the SHV but not the SVV. To our knowledge there are not previous studies reporting on the stability of SHV estimates over time when roll-tilted. The results from the SHV paradigm therefore raise new questions, especially, to which extent paradigm-related factors influence drift in perceived direction of gravity. 

### Estimates of earth-vertical immediately after prolonged roll-tilt are biased towards the previous roll position

The decay dynamics of the post-tilt SVV bias were significantly better fit by an exponential function than a linear function, confirming previous [25,26]. Noteworthy, the additional free parameter in the exponential function likely contributed to the improved fitting relative to using the linear function. However, with the duration of the decay time constant being clearly briefer than the duration of SVV recording (5min), this supports an exponential rather than a linear drift behavior. Compared to the roll-tilt conditions, the median time constant in the post-tilt conditions was considerably shorter (71sec vs. 121sec). Re-adaptation, i.e. return to normal (earth-vertical in our case) was noted to be faster than adaptation away from normal in other paradigms as in certain saccadic gain adaptation paradigms [66], being consistent with our findings. Due to the post-tilt bias, spatial orientation when back upright is initially impaired, gaining accuracy gradually over several minutes. During this time balance or object manipulation might be compromised. 

In order to explain the post-tilt bias, we propose a shift of the reference position (‚null position’) of the gravity estimating system towards the body-longitudinal axis. Our findings suggest a significant contribution of prior knowledge to spatial orientation when changing the roll position (e.g. returning back upright) after prolonged static roll-tilt. In order to improve internal estimates of direction of gravity, the brain takes prior knowledge about head roll into account, assuming that small roll angles are most likely [6,31]. As the prior probability distribution is susceptible to changes in the recent past [67,68], the post-tilt bias can be interpreted as the downside of the brain’s strategy to implement prior knowledge when estimating the direction of gravity. 

Previously, Day and Wade reported a post-tilt bias of similar amplitude for head-on-trunk roll independently of the subject’s orientation relative to gravity (upright vs. supine position) [26,69]. Based on this finding, they concluded that adaptation of the otolith organs is unlikely a relevant factor and proposed that adaptation of neck or trunk receptors contribute to the post-tilt bias [25]. As our experimental setup required the head and the trunk being aligned all the time, adaptation of neck receptors was not the case. However, the adaptation of other peripheral receptors including joint receptors and skin proprioceptors may have contributed to the post-tilt bias. 

To test the hypothesis whether these drift patterns – at least partially – are based on the same (peripheral or central) mechanisms, we performed a subject-by-subject correlation analysis between the drift while roll-titled and the post-tilt drift. We did not find such a correlation, which leads us to the conclusion that the drift during prolonged roll-tilt and the post-tilt bias are not emerging from a single adaptation phenomenon, but distinct mechanisms may influence one or both of them. Specifically, the lack of correlation suggests that the post-tilt bias does not depend on the inaccuracy of the roll-tilt estimate while tilted. Instead, it is rather the previous head-roll orientation relative to gravity that defines the post-tilt bias. Whether the post-tilt bias is based solely on central adaptation mechanisms (e.g., a shift in prior knowledge towards the previous roll orientation due to prolonged roll-tilt or a widening of the prior probability distribution) or whether adaptation of distinct peripheral sensors also contributes, cannot be concluded based on our data set. The high degree of symmetry of the bias (i.e. significant error decrease immediately after both prolonged RED or LED roll) noted in the post-tilt trials suggests a systematic and individually consistent shift in the gravitational null, probably dominated by central adaptation. Re-adaptation of the distribution of prior knowledge and potentially also of peripheral sensors will subsequently lead to an exponential decay of the post-tilt bias. The neuronal networks, which drive such a shift in prior knowledge, have not been identified so far. Current knowledge suggests that temporo-parietal areas are involved in multisensory integration of visuo-vestibular signals [1]. Therefore, these cortical areas may also be involved in the emergence of the post-tilt bias.

### Comparison of drift amplitudes with values from previous studies

The interpretation of differences in the results in our study compared to previous work reporting on SVV drift must be made with caution for several reasons: 1) while subjects were seated upright and were roll-tilted along a dorso-ventral axis in our experiment, they either were rolled along a dorso-ventral axis while standing [14] or along a cranio-caudal axis while being in prone position with the head extended relative to the trunk [12,13,15] in previous studies. The different body-positions may have an impact on the proprioceptors and therefore on drift properties. 2) While we subcategorized individual subjects based on their drift patterns, which had an impact on the amplitude and direction of drift, this has not been done by other groups.

When pooling the SVV data from all subjects, median drift amplitudes were not significantly different from zero and had values of 1.6° or less (±45° roll tilt during 5min) and 4.7° or less (±90° roll tilt during 5min). These median drift values are smaller than the mean SVV drift values over eight minutes of static roll-tilt at ±45° (about 3° of drift, [13]) and ±90° roll-tilt (about 10° of drift, [12,13]) reported by others. Our merged data therefore suggests that the SVV is fairly stable over five minutes of roll-tilt; contrary to the population drifts reported previously. Expanding the data analysis to a single subject level, however, we observed a wide range of sometimes diverging drift patterns at different roll angles. Pooling of individual traces therefore pretends a stability of the SVV during prolonged roll-tilt, which does not reflect the significant drifts observed in 74% of runs. This level of data analysis was not provided in previous studies, allowing no comparison with our results. Taken together, we do agree with previous authors that the SVV may be subject to drift, however, emphasize individual differences in drift amplitude and direction, which might not be depicted when restricting the data analysis to the group level. 

The size of the median absolute post-tilt bias noted here (including all 29 subjects) after prolonged whole-body roll-tilt of ±45° and ±90° was larger (2.4°) than the mean value (1.1°) reported by Day and Wade [25,26]. Both the smaller roll-tilt angle (±30°) and the shorter duration of the roll-tilt period (2min instead of 5min) in the study by Day and Wade may explain the smaller post-tilt bias. 

## Conclusions

The lack of correlation between the drift pattern while roll-tilted and the bias observed upon return to upright suggests that distinct peripheral and central mechanisms contribute to these two phenomena. Specifically, it is not the inaccuracy of the SVV estimate while tilted that determines post-tilt bias, but rather the previous head-roll orientation relative to gravity. This leads us to the conclusion that for the post-tilt bias central adaptive mechanisms (i.e. of prior knowledge) are probably most important. However, peripheral adaptation (e.g. of proprioceptive receptors) cannot be excluded. Drift while roll-tilted, on the other hand, is likely related to both adaptation of peripheral sensors (including torsional eye position) and central integrative networks potentially including a change in the prior probability distribution, while random-walk effects and direct motor effects of ocular torsion seem to be minor contributors. In future SVV studies that include prolonged roll-tilt, both the individually distinct drift and the bias when returning back upright should be taken into consideration; grouping of subjects and a break with the lights turned on upon return to upright position may minimize the transient post-tilt bias and avoid misinterpretation of experimental findings. 
